# Missed or Delayed Diagnosis of Heart Disease by the General Pediatrician

**DOI:** 10.3390/children12030366

**Published:** 2025-03-15

**Authors:** Ageliki A. Karatza, Sotirios Fouzas, Despoina Gkentzi, Eirini Kostopoulou, Christina Loukopoulou, Gabriel Dimitriou, Xenophon Sinopidis

**Affiliations:** 1 Department of Pediatrics, University Hospital, School of Medicine, University of Patras, 26504 Patras, Greece; karatza@upatras.gr (A.A.K.); sfouzas@upatras.gr (S.F.); gkentzid@upatras.gr (D.G.); irekost@upatras.gr (E.K.); christina.loukopoulou@upatras.gr (C.L.); gdim@upatras.gr (G.D.); 2 Department of Pediatric Surgery, University Hospital, School of Medicine, University of Patras, 26504 Patras, Greece

**Keywords:** heart disease, diagnosis, congenital heart disease

## Abstract

Missed or delayed heart disease diagnoses pose a major challenge in pediatric primary care. Many cardiac conditions present with subtle or nonspecific symptoms that resemble benign childhood illnesses, making their prompt recognition difficult. This review describes congenital and acquired heart diseases prone to diagnostic delays, including critical congenital heart disease, coarctation of the aorta, atrial and ventricular septal defects, myocarditis, Kawasaki disease, heart failure, and pulmonary arterial hypertension. The atypical presentations of these disorders and the associated diagnostic pitfalls are emphasized. Furthermore, the importance of alarming symptoms and signs, such as chest pain, palpitations, syncope, and abnormal heart murmurs, is underscored. A structured approach to these red flags is presented to assist primary care pediatricians in identifying children at risk, initiating appropriate management, and referring them for specialized evaluation. The importance of preparticipation screening for athletes is also discussed, highlighting how it can be applied to all children during routine health visits to identify those with heart disease. Appropriate training is essential to increase pediatricians’ ability to recognize and manage cardiac patients.

## 1. Introduction

Heart disease in children becomes manifest at different ages, with clinical presentation ranging from incidental auscultation findings to cyanosis, congestive heart failure (HF), or cardiovascular collapse, depending on the anatomy and physiology of the lesion [1,2,3]. Congenital heart diseases (CHDs), albeit relatively rare, may be associated with a high disability risk and require life-long specialized medical care, even in its milder forms [4,5,6,7,8]. For instance, aortic bicuspid valve (the most common CHD in all ages with a prevalence of 0.5 to 2%) often goes unnoticed during childhood and adolescence, but typically manifests complications in adulthood [5]. On the other hand, acquired heart diseases are gaining increasing attention due to their substantial burden, which may be comparable to that of CHD [9,10,11]. The two most common conditions are rheumatic heart disease (in developing countries [12,13]) and Kawasaki disease (in the developed world [14]), followed by myocarditis, pericarditis, endocarditis, and cardiomyopathies [15]. All require prompt diagnosis and treatment to prevent severe complications and avoid long-term sequelae [15].

Primary care pediatricians play a pivotal role in the medical care of children and adolescents, facing the challenge of identifying and addressing significant clinical concerns within a typically healthy population. Thus, the diagnosis of a heart disorder may be delayed or missed, especially when it is expressed with subtle signs and symptoms that mimic benign childhood illnesses [16,17,18,19,20,21]. Moreover, heart diseases with very low prevalence may remain undiagnosed due to general pediatricians’ limited familiarity and lower levels of suspicion [18,19]. In a study in Switzerland, late diagnosis was observed in 10% of the total population, 6% of cyanotic CHD patients, and 13% of non-cyanotic CHD patients [22]. In developing countries, delayed or missed diagnosis has been reported for Kawasaki disease (KD), myocarditis, heart failure (HF), coarctation of the aorta (CoA), and hypoplastic left heart syndrome; the misclassification of heart murmurs is also very common [23,24].

In this narrative review, we present heart conditions that could potentially result in delayed or missed diagnoses by the general pediatrician. Drawing from recent literature and key European and American guidelines, we provide readers with essential information to recognize the red flags of cardiac diseases that may resemble benign childhood illnesses, and understand the critical clinical points to be reviewed when screening healthy children for cardiac defects.

## 2. Congenital Cardiac Disorders Prone to Delayed or Missed Diagnosis

### 2.1. Critical CHD

Critical CHDs represent a group of potentially life-threatening disorders; they account for 30% of all CHD cases and are defined as conditions needing intervention or resulting in death within one month after birth [25,26]. Delayed diagnosis is associated with cardiovascular compromise and organ dysfunction, resulting in poor surgery outcomes and increased morbidity and mortality [27,28,29].

Most newborns with critical CHD are asymptomatic at birth [29]. Screening for CHD typically involves a second-trimester ultrasound scan and postnatal clinical examination of the cardiovascular system; a postnatal echocardiogram should be performed if the latter yields abnormal findings [30,31]. Second-trimester ultrasound screening, however, has variable and often low detection rates [32]. A meta-analysis of seven relevant studies showed a detection rate of CHD in unselected populations of about 45% [30]. Moreover, the persistence of fetal circulation after birth can mask the clinical presentation of CHD. Thus, critical clinical signs—such as heart murmurs and weak or delayed femoral pulses—are often absent in the early postnatal period, while mild cyanosis frequently goes undetected in the first days of life [33].

In 2011, the American Academy of Pediatrics (AAP), the American College of Cardiology Foundation, and the American Heart Association (AHA) found sufficient evidence to suggest universal screening for low blood oxygen saturation by pulse-oximetry to detect ductal-dependent CHD in newborns admitted in well-infant nurseries [31,34]. Screening is recommended in all healthy neonates when they are at least 24 h of age, or as late as possible if they are to be discharged earlier than 24 h [31,35]. However, earlier screening may be associated with false-positive results [34]. In conjunction with antenatal fetal ultrasound and postnatal physical examination, pulse oximetry screening significantly improves (i.e., almost doubles) the detection rate of critical congenital heart defects [26].

### 2.2. Coarctation of the Aorta

The coarctation of the aorta occurs in 0.3 per 1000 live births, accounting for 6–8% of all CHDs [36]. Most cases fall into one of the following categories: (a) critical CoA (60% of cases) that becomes manifest in the neonatal period when the arterial duct closes and which, if left untreated, is lethal, and (b) asymptomatic CoA that presents later in childhood, usually with arterial hypertension in the upper limbs [37].

Critical CoA in neonates is a common cause of shock and death. It may be the most difficult of all forms of critical CHD to diagnose, because there are no characteristic murmurs, and the obstruction does not appear until several days after birth (i.e., after discharge from the hospital) when the arterial duct closes [36,37,38]. Infants with CoA typically become symptomatic between 7 and 14 days after birth, and they are often misdiagnosed as having sepsis [37,38,39]. Decreased femoral pulses and upper limb hypertension are usually present before this event, underlying the importance of regularly checking brachial and femoral pulses throughout the first month of life [40]. Although the arterial duct presents right-to-left flow before closure, pulse oximetry has low sensitivity in detecting CoA in the early postnatal period [41,42].

The diagnosis in older children is based on identifying hypertension in the arms, weak and delayed femoral pulses, and a systolic or continuous murmur in the interscapular area or at the left border of the spine [39,43]. Of note, blood pressure should be taken with an appropriate-sized cuff in both arms because the left subclavian artery is often hypoplastic or distal to the coarctation, while the right subclavian artery may be aberrant in more than 1% of the population and, thus, could originate peripherally in the coarctation site [44,45]. If the arm pressure is high and associated with weak and delayed femoral pulses, the next step is obtaining leg blood pressure with an appropriate-sized cuff [44].

In older children, CoA escapes diagnosis in about 85% of cases, despite the characteristic clinical signs [37]. In a study from the US, the median age at diagnosis was 10 years, and the diagnosis was made before referral in only 14% of the patients [46]. The remaining referrals had been made after the incidental notation of hypertension or heart murmur [46]. In a study performed at the same institution 14 years later, the mean age of diagnosis was 8.4 years [47]. The findings are similar to those reported in the previous decade, suggesting that the early detection of CoA was not improved with time [47].

### 2.3. Secundum Atrial and Ventricular Septal Defects

Secundum atrial septal defects (ASDs) have an incidence of 230 per 10,000 live births [48]. There are five types of ASDs: patent foramen ovale, ostium secundum defect, ostium primum defect, sinus venosus defect, and coronary sinus defect [49].

The diagnosis is challenging. The physical examination features of a wide fixed split S2, systolic pulmonary flow murmur, or diastolic rumble across the tricuspid valve can be subtle, and the electrocardiographic features (right axis deviation, right bundle branch block, right atrial enlargement, or right ventricular hypertrophy) may be absent [49,50]. The systolic pulmonary flow murmur may resemble an innocent pulmonary flow murmur; the diastolic murmur is also soft and may not be auscultated, while the detection of a widely fixed split S2 (usually the only abnormal auscultation feature) is difficult in an infant or child with a rapid heart rate [50]. A study from the Boston Children’s Hospital revealed that 37% of the patients were diagnosed in the first year and 72% in the first decade of life [50]. The detection of a murmur was the most common feature prompting initial evaluation, while the rest of the diagnoses were achieved by an incidental echocardiogram [50].

Patients with defects under 5 mm might not develop any symptoms; those with ASDs ranging from 5 to 10 mm will present in the fourth or fifth decade of life, while patients with more significant defects will present sooner [49]. Untreated large defects can cause exercise intolerance, dyspnea, fatigue, palpitations, and signs of right-sided heart failure, and are associated with an increased risk of stroke or transient ischemic attack, pneumonia, and pulmonary hypertension in adulthood [51]. 

Ventricular septal defects (VSDs) are present in 42 per 10,000 live births [48]; they may be conoventricular, perimembranous, inlet ventricular, or muscular, and appear as isolated lesions or as components of complex CHDs [52]. Because lung blood flow increases after birth, larger VSDs tend to manifest symptoms within four to six weeks in term infants, or within the first two weeks of life in preterm infants [52,53]. Symptoms include failure to thrive, increased work of breathing, tachypnea, and respiratory distress [52]. If the defect is small and hemodynamically restrictive (i.e., with limited size of the left-to-right shunt), the patient is asymptomatic, and the condition is discovered incidentally by the presence of a murmur [52,54].

In patients with large VSDs but low pulmonary resistance, the precordium is hyperactive, and the murmur is harsh and holosystolic. A diastolic rumble heard in the mitral area (due to functional mitral stenosis) and/or an ejection systolic murmur (due to increased pulmonary flow) confirm the presence of a large VSD [52,54]. When the defect is hemodynamically restrictive, and the left ventricular pressure is greater than the pressure in the right ventricle, the murmur is typically loud, harsh, and often associated with a thrill. If the defect begins to close spontaneously, the murmur becomes attenuated [52]. Infants with a muscular VSD frequently have no heart murmur [53]. A study from the Czech Republic showed that VSD and CoA constituted the majority of delayed diagnoses despite the presence of a murmur [55].

The prenatal echocardiographic diagnosis of VSDs is also challenging. The sensitivity of detection is highly variable and depends on operator experience, gestational age, and the position of the fetus [56]. A multicenter study from China showed that perimembranous VSDs were more easily diagnosed prenatally, but with a lower success rate than other CHDs [56].

## 3. Acquired Cardiac Disorders Associated with Delayed or Missed Diagnosis

### 3.1. Myocarditis

Myocarditis affects children of all ages, although most cases occur in infants and adolescents. More than half of those diagnosed with myocarditis are less than one year old [46]. Its impact on childhood morbidity and mortality is significant: it accounts for nearly 30% of all cases of dilated cardiomyopathy and has been identified as a cause of sudden, unexpected death in young patients [57,58,59]. Myocarditis in children is mainly of infectious (viral) etiology [58,59]. Vaccine-associated myocarditis has also been reported in adolescent males after the second dose of the mRNA vaccine against COVID-19, although the association is highly debatable and still unclear [60]. The exact incidence of myocarditis in childhood is difficult to estimate due to its variable clinical presentation miming other, more common pediatric diseases [57,58,59].

Children with myocarditis may present non-specific symptoms that can be mistaken for other types of illnesses (e.g., of upper respiratory or gastrointestinal tract) [57,58,59]. The clinical characteristics of myocarditis may involve malaise, fatigue, anorexia, shortness of breath, fever, rhinorrhea, mild chest pain, orthopnea, presyncope, dyspnea, cough, palpitations, abdominal pain, vomiting, and diarrhea, depending on the age of the child [57,59]. The history of a preceding viral prodrome is present in about two-thirds of cases [57,58,59]. Fever has been reported in >50% of patients [58]. Arrhythmias are common and include ventricular, atrial arrhythmias, and high-grade atrioventricular block [57,58,59]. Syncope occurs in about 10% [58]. Acute severe myocarditis presents with heart failure, cardiovascular collapse, or sudden cardiac death [57,58,59]. Most children admitted with myocarditis have multiple visits to medical providers before being correctly diagnosed [57]. Many patients may be initially considered as having either sepsis, pneumonia, or asthma, while those presented with nausea and vomiting may be misdiagnosed as having gastroenteritis [57,61].

Prompt diagnosis requires a high level of suspicion and is based on clinical signs, electrocardiography, serum levels of cardiac troponin I, creatine kinase-MB and N-terminal B-type natriuretic peptide, and echocardiographic findings (decreased ventricular ejection fraction, valvular regurgitation, uncoordinated or weakened ventricular wall motion) [57,58,59,62]. Currently, cardiac magnetic resonance imaging (CMRI) has emerged as a powerful diagnostic and monitoring tool. Now, CMRI has largely replaced endomyocardial biopsy, which is invasive and reserved for more complex cases of pediatric myocarditis. However, CMRI is not easily accessible, and often requires general anesthesia or sedation for the pediatric age group [57,58,59,62].

### 3.2. Kawasaki Disease

Kawasaki disease is a self-limited acute vasculitis of the medium caliber muscular arteries of unknown etiology. It predominantly affects children under five years of age and represents the most common cause of acquired pediatric heart disease in the developed world [11,63]. Classic KD is diagnosed in the presence of fever for at least five days together with four of the following clinical features [11,63]: (a) bilateral conjunctival injection without discharge, (b) erythema and cracking of lips, strawberry tongue, and/or erythema of oral and pharyngeal mucosa, (c) rash—maculopapular, diffuse erythroderma, or erythema multiforme-like, (d) erythema and edema of the hands and feet in acute phase and/or periungual desquamation in subacute phase, and (e) cervical lymphadenopathy (≥1.5 cm diameter), usually unilateral. The most important complications are coronary artery aneurysms, which are seen in up to 25% of untreated cases [11,63]. Early treatment (within 10 days of illness) with intravenous immunoglobulin decreases their incidence to 4% [11].

The diagnosis of incomplete or atypical KD should be considered in any child with prolonged unexplained fever, less than four of the principal clinical criteria, and compatible laboratory or echocardiographic findings [63]. Incomplete Kawasaki is also characterized by a younger age of onset, a higher incidence of coronary artery aneurysms, enhanced immune tolerance to immunoglobulin, and a delayed immune response compared with KD [11,64].

Evaluation with laboratory investigations for suspected incomplete KD should be performed in children with fever for ≥5 days and two or three clinical criteria or infants with fever for ≥7 days without another explanation [11]. C-reactive protein ≥ 3 mg/dL and erythrocyte sedimentation rate ≥40 mm/h, if associated with three or more abnormal laboratory findings, such as anemia for the age of the child, platelets ≥ 450,000/μL after the seventh day of fever, albumin ≤ 3 gr/dL, increased alanine aminotransferase, white blood cell count ≥ 15,000/mm^3^, ≥10 white blood cells/high-power field in the urine, and an abnormal echocardiogram identify the candidates for treatment [11].

The higher prevalence of coronary artery lesions in atypical KD is the result of delayed diagnosis and treatment [11,64,65,66]. A lack of awareness and delays in management have been documented in pediatric clinical practice; children with suspected KD should be referred for more specialized evaluation and management [11,64,66,67].

### 3.3. Heart Failure

Heart failure (HF) has an incidence of 0.87 per 100,000 children in the United Kingdom and Ireland and 7.4 per 100,000 in Taiwan [68]. In the developed world, CHD and cardiomyopathies are the leading causes, followed by arrhythmias and acquired heart diseases, such as myocarditis and dilated cardiomyopathy [68].

Due to the rarity of HF in the pediatric population, most practitioners have little experience with its presentation and management [69]. Prompt recognition is further complicated by its marked heterogeneity in the underlying causes, pathophysiology, and clinical presentation [70]. Indeed, half of the children with unknown heart disease who are hospitalized with systolic HF are not identified at first, and undergo irrelevant treatment and investigations [19,71]. Initial presentation to a primary care physician, longer duration of symptoms, and non-specific clinical presentation (i.e., gastroenterological symptoms in older children) have been associated with missed diagnosis [19].

The clinical presentation of HF in children can be quite variable, leading to a considerable overlap with more common conditions like asthma, pneumonia, and gastroenteritis [72]. Its timely diagnosis depends on a high suspicion index and the thoughtful use of diagnostic studies [69,71]. Respiratory symptoms such as cough, shortness of breath, increased work of breathing, and tachypnea are seen in most of the patients [24,69,71]. Abdominal pain, nausea, vomiting, decreased appetite, weight loss (in older children), feeding intolerance, and failure to thrive (in infancy) are also common [69,73,74]. Gastroenterological symptoms are more prevalent in adolescents, whereas respiratory complaints are more common in younger children and infants [74]. When present, hepatomegaly can be helpful as it is uncommon in children and more specific to HF. Resting tachycardia and tachypnea are commonly present in all ages [74]. Hepatomegaly or cardiomegaly (in imagistic studies) and an abnormal electrocardiogram (ECG) may help distinguish HF from other more common pediatric illnesses [62,71,72,73,74].

### 3.4. Pulmonary Arterial Hypertension

Pulmonary arterial hypertension (PAH) is characterized by increased pressure in the pulmonary arteries caused by a heterogeneous group of diseases with substantial morbidity and mortality [75,76,77,78,79,80,81]. It is a rare entity in childhood, and as with any rare disease, there is an increased risk of delayed or missed diagnosis [75,76,77,78,79,80].

The most common symptoms are dyspnea on exertion, fatigue, and syncope [75,76,79]. Symptoms are less specific in infants and may involve poor appetite, failure to thrive, diaphoresis, tachypnea, tachycardia, and irritability [75,76,79]. Children with pulmonary arterial hypertension are often initially misdiagnosed as having more common childhood conditions, such as asthma, vasovagal syncope, or seizures [78].

A standardized approach to diagnosing PAH has been recently recommended by the European Society of Cardiology/European Respiratory [75]. The diagnostic approach is mainly focused on (a) raising early suspicion of PAH and ensuring fast-track referral to specialized centers, and (b) identifying the underlying cause [75]. Screening infants with bronchopulmonary dysplasia for PAH is also recommended [75]. The determination of pulmonary artery pressure by Doppler echocardiography is central to patient screening and evaluation [82]. For confirming PAH, right heart catheterization is recommended before initiating any therapy [75,83,84].

### 3.5. Chemotherapy-Induced Cardiotoxicity

Chemotherapeutic drugs may have detrimental effects on the heart and the circulatory system [85]. Cardiovascular disease is the leading contributor to morbidity and mortality in pediatric cancer survivors in the long term [86,87]. Chemotherapy-induced cardiotoxicity may appear acutely, early, or late [85]. The acute type is rare, occurs within one week of therapy, presents as ventricular dysfunction, and is fully reversible with the removal of the inciting agent. The early onset (subacute) type occurs within one year of chemotherapeutic exposure. It is characterized by left ventricular dilation and depressed contractility, and its course is progressive. Late-onset (chronic) cardiotoxicity occurs more than one year out from first exposure, mimics dilated cardiomyopathy (often with additional restrictive features), and is also progressive [85].

## 4. Alarming Symptoms and Signs

The unique cardiovascular physiology of heart disease in children, which is attributed to changes in growth and development from birth to adulthood, is associated with differences in presenting signs and symptoms of heart disease compared to adults [22]. Primary care pediatricians play a crucial role in diagnosing, initiating management, and referring patients for specialized cardiology evaluation [88,89,90] by recognizing and assessing key warning signs and symptoms [Table 1].

During routine well-child visits, a detailed medical history, including cardiovascular symptoms such as chest pain, syncope, palpitations, exercise intolerance, and feeding difficulties, as well as a family history of heart disease in close relatives, should be obtained [91,92]. Careful physical examination can be a useful screening tool in pediatric office settings [91,92], particularly when access to echocardiography is limited [93]. Red flags in personal history include in-utero exposure to cardiac teratogens, feeding difficulties, and failure to thrive in neonates and infants. In older children and adolescents, concerning signs include respiratory symptoms, cyanosis, frequent lower respiratory infections linked to left-to-right shunting, chest pain, syncope, and exercise intolerance [94,95,96].

Alarming findings from physical examination include syndromic features, failure to thrive, peripheral edema, hyperdynamic precordium, delayed and weak femoral pulses, abnormal S2, intense (≥3/6) cardiac murmur, harsh quality of the murmur, a systolic click, a diastolic or holosystolic murmur, increased intensity of the murmur in standing position suggestive of hypertrophic cardiomyopathy, ascites, and hepatomegaly [95,96,97]. It should be noted, however, that only 1% of heart murmurs represent cardiac pathology [94]. Distinguishing murmurs caused by an underlying heart defect from those caused by blood flow within a structurally normal heart can be challenging for the primary care physician, and requires significant clinical skills [95].

Chest pain in children and adolescents is common, but—as opposed to adults—is rarely (0.2–1%) of cardiac etiology [98,99,100]. Nevertheless, the symptom usually leads to costly and unnecessary investigations, medical visits, and hospitalizations [100]. Chest pain in children may be of musculoskeletal, pulmonary, gastrointestinal, or psychogenic origin [96]. Idiopathic chest pain is the most common and characterized by typically chronic or relapsing symptoms with repeatedly normal laboratory testing [99]. Cardiac causes include aortic dissection, pericarditis, myocarditis, congenital and acquired coronary abnormalities, cardiomyopathies, severe aortic or subaortic obstruction, and arrhythmias [99,100]. Primary care physicians can be reassured that cardiac pathology is excluded when the patient’s personal and family history, physical examination, and ECG are normal [100,101,102]. Red flags include chest pain with physical activity, pain associated with palpitations or syncope, family history of sudden cardiac death or cardiomyopathy in first-degree relatives, known history of CHD or KD, and chest pain associated with electrocardiographic abnormalities [100,101,102].

Palpitations are defined as perceived abnormalities in the heartbeats described by the patient as bouncing, fast, or irregular. The most common diagnoses are supraventricular tachycardia and premature atrial or ventricular contractions [103]. Elements from the medical history suggestive of clinically important palpitations are abnormal beats that appear and disappear abruptly, a “too fast to count” heart rate, palpitations that occur at exercise, and palpitations associated with chest pain, shortness of breath, dizziness, or syncope. It is important to document whether palpitations last for seconds or hours, and if they occur often or occasionally [104]. The ECG may record the arrhythmia or identify pre-excitation that places the patient at risk for arrhythmias. A Holter monitor is not useful in evaluating infrequent episodes of palpitations, but will determine their cause if the frequency of ectopy is high [103].

Syncope is the transient loss of consciousness and postural tone resulting from an abrupt, temporary decrease in cerebral blood flow [104,105]. Approximately 15% of children will experience at least one episode up to the age of 18 years [105], making syncope one of the most common referrals to pediatric cardiology and neurology clinics [103,104]. The child’s age is important, as it is a rare phenomenon in preschoolers except for breathing-holding spells [104]. The non-life-threatening types of syncope include reflex syncope and orthostatic hypotension. Reflex syncope is the most common and is attributed to reflex (vagal) nervous hyperactivity that results in a slow heart rate and hypotension [103]. Orthostatic hypotension represents an impairment of systemic vascular resistance due to various causes concerning the autonomic nervous system [106]. Features suggestive of reflex syncope include occurrence after an emotionally stressful event, after prolonged standing in a hot or crowded area, during or after a meal, exercise, or head rotation. Prodromal symptoms, such as nausea and vomiting, may also exist [105]. Syncope due to orthostatic hypotension occurs following standing from a supine, sitting, or squatting position, standing after exercise, prolonged standing in a hot environment, and secondary to the commencement of vasoactive medications [106]. The serious, life-threatening causes of syncope are generally cardiac in nature, such as arrhythmia and structural heart disease [103,106]. Red flags included syncope with no prodromal symptoms, syncope during exertion, family history of sudden unexplained death in first-degree relatives, abnormal ECG, a systolic murmur that intensifies with the Valsalva maneuver, gallop rhythm, and unexplained tachycardia [103]. A thorough personal and family history, a detailed physical examination, and an ECG are sufficient to avoid unnecessary and expensive diagnostic investigations [103,104,105,106,107]. The ECG allows screening for dysrhythmias, such as Wolff–Parkinson–White syndrome, heart block, Brugada pattern, long QT syndrome, hypertrophic cardiomyopathy, and myocarditis [103]. Patients presenting with signs or symptoms attributed to heart disease should be restricted from physical exercise and be referred for pediatric cardiological evaluation. Children with exertional syncope should undergo evaluation with an echocardiogram and exercise testing [104,107].

## 5. Implementing Structured Screening

While much focus has been placed on preventing sudden cardiac death (SCD) in young athletes, efforts have also been made to screen the general pediatric population for potentially serious cardiac conditions [97,108,109]. Thus, according to the AAP, the preparticipation screening for athletes should be applied to all children [110]. Common pathologic conditions that put children at risk for SCD include cardiomyopathies, channelopathies (long QT syndrome, short QT syndrome, Brugada syndrome, catecholaminergic polymorphic ventricular tachycardia and idiopathic ventricular fibrillation), congenital heart disease, Wolf–Parkinson–White syndrome, anomalous coronary arteries and aortopathies [110].

The AHA recommends 14 key elements during preparticipation examinations to identify children at risk, as follows [111]:

(1) Chest pain, discomfort, tightness, or pressure during exercise;
(2) Unexplained syncope or near-syncope not attributed to a vasovagal cause;
(3) Excessive or unexplained dyspnea, fatigue, or palpitations during exercise;
(4) Personal history of heart murmur;
(5) Elevated arterial blood pressure;
(6) History of sports participation restrictions due to medical concerns;
(7) Previous cardiac testing ordered by a physician;
(8) Family history of premature cardiac-related death before age 50;
(9) Disability due to heart disease in a close relative under age 50;
(10) Family history of hypertrophic or dilated cardiomyopathy, long QT syndrome, other ion channelopathies, Marfan syndrome, clinically significant arrhythmias, or known genetic cardiac conditions;
(11) Identification of a heart murmur, not felt to be innocent;
(12) Unpalpable, decreased or delayed femoral pulses;
(13) Physical stigmata of Marfan syndrome;
(14) Increased brachial artery blood pressure (sitting position, both arms).

A positive response to any of these points warrants specialized cardiovascular evaluation [111].

According to the AAP, all children should be asked during a routine health visit the following [110]:

(1) If they have ever fainted, passed out, or experienced an unexplained seizure suddenly and without warning, particularly during exercise or in response to sudden loud noises, such as doorbells, alarm clocks, or ringing telephones;
(2) If they have ever experienced chest pain or shortness of breath during exercise;
(3) If they have anyone in their immediate family (parents, grandparents, or siblings) or in the extended family (aunts, uncles, or cousins) who died from heart problems or suffered an unexpected sudden death before the age of 50;
(4) If they have relatives with hypertrophic obstructive cardiomyopathy, Marfan syndrome, arrhythmogenic cardiomyopathy, long QT syndrome, short QT syndrome, Brugada syndrome or polymorphic ventricular tachycardia, or relatives under the age of 50 who have a pacemaker or implantable defibrillator.

A positive response to one of the above questions should prompt further investigation by a pediatric cardiologist [110].

The European Society of Cardiology, in addition to history and physical examination, recommends screening with ECG for all young athletes involved in competitive sports [112]. Thus, ECG is generally part of pre-participation evaluation in European countries [113]. Conversely, such massive screening with ECG has been regarded as complex, and is not routinely performed in the US [111]. A recent meta-analysis concluded that the odds of detecting conditions related to SCD are greater with ECG than with history and physical examination alone [113]. The ECG should be the first test ordered when there is a concern for SCD, and should be interpreted by a trained physician [112,113]. The computer analyzer of the ECG should not be used, as it is unreliable in children [110].

## 6. Improving Pediatric Training

In the current era of increasing specialized care, standardized pediatric training on cardiology topics is paramount to optimizing outcomes. Pediatric trainees are likely nowadays less experienced in managing ill cardiac patients, as they may not rotate through specialized cardiac care facilities during internship [114]. Meanwhile, as medical knowledge rapidly expands, educators must cover an increasing volume of material, reducing the time available to focus on each pediatric subspecialty. Moreover, advances in prenatal diagnosis and universal neonatal screening by pulse oximetry decrease the opportunities for trainees to be exposed to the diagnostic challenge of an unknown CHD [115,116].

Pediatric residents must be able to diagnose, triage, and manage infants and children with CHD, and the American Board of Pediatrics has issued specific guidelines for their training on pediatric cardiology topics [117]. However, since the teaching on this subspecialty service varies and may depend on the patient volume, residents may not be exposed to all topics during their pediatric cardiology rotation [118]. A single-center study showed that 76% of pediatric residents were less than very comfortable with the differential diagnosis of cyanosis in a newborn, and 92% were less than very comfortable with murmur identification [119]. Revised training curricula featuring shorter lectures and increased active learning through faculty and peer engagement significantly increase residents’ ability to recognize and manage pediatric cardiac patients [119,120,121].

## 7. Limitations

This narrative review is mainly based on data from developed countries regarding missed or delayed diagnoses of pediatric heart disease. There is little reliable information on the spectrum and prevalence of congenital and acquired cardiac disorders in children in low-income countries, but enough to know that the burden is considerable, with patients typically presenting with advanced disease due to a shortage of financial sources, limited trained medical and nursing personnel, and barriers to accessing medical facilities [23,93,122,123,124,125]. In a study from Indonesia, the incidence of delayed CHD diagnosis was 60.8%, while it was 54.9% for the cyanotic and 86.2% for the non-cyanotic forms. Delayed diagnosis by the physician was the most common cause, followed by delayed diagnosis related to midwifery care, financial barriers to referral or follow-up, and social factors [24]. Nevertheless, the stepwise evaluation of symptoms and signs, along with the structured screening methods presented here, can universally help primary care pediatricians in preventing delayed and missed diagnoses of pediatric heart diseases.

## 8. Conclusions

Primary care pediatricians often encounter a range of cardiac conditions, with symptoms that vary significantly and may not have an apparent connection to the underlying pathology. Thus, the risk of diagnostic errors or delayed recognition of pediatric heart disorders may be substantial. Nevertheless, the available data remain limited, providing little insight into the most frequently misdiagnosed conditions or the diagnostic processes most susceptible to error. This narrative review highlights the atypical presentation of congenital and acquired pediatric heart disorders, which can lead to missed or delayed diagnoses. Additionally, it emphasizes the importance of a structured clinical approach, including personal and family history and thorough clinical examination. Common symptoms and complaints potentially linked to heart disease are outlined, along with key red flags that should prompt further cardiac evaluation. Primary care pediatricians must be equipped to promptly identify, triage, manage, and refer children with cardiac disease, underscoring the critical importance of proper training.

## Figures and Tables

**Table 1:** Assessment of alarming symptoms and signs.

Symptom or Sign	Etiology	Red Flags	Investigations
Chest pain	Non-cardiac: musculoskeletal conditions (costochondritis, Tietze’s Syndrome, idiopathic chest-wall pain, trauma, muscle strain), respiratory (asthma, lung and pleural infections, pneumothorax, pneumomediastinum), skin lesions (herpes zoster), psychogenic Cardiac: pericarditis, myocarditis, severe pulmonary or aortic valve stenosis, anomalous coronary arteries, supraventricular tachycardia, premature ventricular complexes, ventricular tachycardia, hypertrophic obstructive cardiomyopathy	History: family history of sudden death; personal history of Kawasaki disease, diabetes mellitus, or heart surgery; accompanying symptoms such as syncope, palpitations, exertional chest pain; drug abuse Physical examination: poor peripheral pulses or perfusion, upper body hypertension, delayed femoral pulses, hepatomegaly, hyperactive precordium, abnormal heart sounds, murmurs increasing in intensity in the upright position	If clinical features suggest non-cardiac origin (pain with motion/cough, bilateral distribution, non-exertional chest pain): provide reassurance that no further investigations are required If there are suspicions of cardiac origin: chest X-ray, ECG, high-sensitivity troponin assay, and refer for pediatric cardiology consultation (echocardiography, Holter monitoring, cardiac magnetic resonance)
Palpitations	Benign: sinus bradycardia, first-degree atrioventricular block, asymptomatic Mobitz I atrioventricular block, sinus tachycardia, premature atrial contractions, sinus arrhythmia Of concern: Mobitz II and high-grade atrioventricular block, third-degree (complete) heart block, paroxysmal supraventricular tachycardia, premature ventricular contractions, atrioventricular reentrant tachycardia, Wolf–Parkinson–White syndrome	Abnormal ECG	If benign: provide reassurance If uncertain or of concern: refer for pediatric cardiology consultation (echocardiography, Holter monitoring, exercise testing, electrophysiologic study)
Syncope	Benign: neurocardiogenic syncope, postural orthostatic tachycardia, orthostatic hypotension Neurologic: seizures, migraine Cardiac: arrhythmias (long QT syndrome, Brugada syndrome, Wolff–Parkinson–White), left-ventricle outflow obstructive lesions (hypertrophic cardiomyopathy, severe aortic stenosis), abnormal origin of coronary arteries	Syncope on exertion, in the recumbent position, syncope without premonitory signs, recurrent syncopal attacks with exercise, palpitations or chest pain with exercise Personal history of heart disease, family history of long QT syndrome, early sudden death, atypical seizures Murmur that increases in intensity in the upright position Abnormal ECG	If benign: perform ECG and provide reassurance If uncertain or of concern: refer for pediatric cardiology consultation (echocardiography, Holter monitoring, exercise testing, head-up tilt testing, electrophysiologic study, cardiac magnetic resonance)
Heart murmur	Innocent murmurs: vibratory Still’s murmur, pulmonary flow murmur, supraclavicular/brachiocephalic systolic murmur, carotid bruit, aortic flow murmur, venous hum, mammary artery soufflé, physiological peripheral pulmonic stenosis in neonates Pathologic murmurs: structural cardiac lesions	Personal history: feeding difficulties, failure to thrive, respiratory symptoms (cyanosis, frequent lower respiratory infections), chest pain in exercise or syncope, exercise intolerance, in utero exposure to infections or toxic substances Family history: sudden cardiac death, Marfan syndrome, cardiomyopathy, congenital heart disease, maternal diabetes Physical examination: syndromic infant, failure to thrive, hyperdynamic precordium, delayed and weak femoral pulses, upper body hypertension, abnormal S2, intense (≥3/6) murmur/thrill, harsh quality murmur, systolic click, diastolic or holosystolic murmur, increased intensity in upstanding position, hepatomegaly	If clinical features suggest an innocent heart murmur (asymptomatic child; normal heart sounds; murmur is systolic, soft, vibratory or musical in quality; murmur is suppressed when changing position from supine to standing): provide reassurance that no further investigations are required When there are red flags, refer for pediatric cardiology consultation (echocardiography, chest X-ray, ECG, Holter monitoring, cardiac magnetic resonance)

ECG: electrocardiogram.
